# Rare Event Simulation for Non-Markovian Repairable Fault Trees

**DOI:** 10.1007/978-3-030-45190-5_26

**Published:** 2020-03-13

**Authors:** Carlos E. Budde, Marco Biagi, Raúl E. Monti, Pedro R. D’Argenio, Mariëlle Stoelinga

**Affiliations:** 8 Johannes Kepler University, Linz, Austria; 9 University of Birmingham, Birmingham, UK; 10 Formal Methods and Tools, University of Twente, Enschede, The Netherlands; 11 Department of Information Engineering, University of Florence, Florence, Italy; 12 FAMAF, Universidad Nacional de Córdoba, Córdoba, Argentina; 13 CONICET, Córdoba, Argentina; 14 Department of Computer Science, Saarland University, Saarbrücken, Germany; 15 Department of Software Science, Radboud University, Nijmegen, The Netherlands

**Keywords:** Rare event simulation, Dynamic fault trees, System reliability analysis

## Abstract

Dynamic fault trees (DFT) are widely adopted in industry to assess the dependability of safety-critical equipment. Since many systems are too large to be studied numerically, DFTs dependability is often analysed using Monte Carlo simulation. A bottleneck here is that many simulation samples are required in the case of rare events, e.g. in highly reliable systems where components fail seldomly. Rare event simulation (RES) provides techniques to reduce the number of samples in the case of rare events. We present a RES technique based on importance splitting, to study failures in highly reliable DFTs. Whereas RES usually requires meta-information from an expert, our method is fully automatic: By cleverly exploiting the fault tree structure we extract the so-called importance function. We handle DFTs with Markovian and non-Markovian failure and repair distributions—for which no numerical methods exist—and show the efficiency of our approach on several case studies.

### 1 Introduction

Reliability engineering is an important field that provides methods and tools to assess and mitigate the risks related to complex systems. Fault tree analysis (FTA) is a prominent technique here. Its application encompasses a large number of industrial domains that range from automotive and aerospace system engineering, to energy and telecommunication systems and protocols.

**Fault trees.** A fault tree (FT) describes how component failures occur and propagate through the system, eventually leading to system failures. Technically, an FT is a directed acyclic graph whose leaves model component failures, and whose other nodes (called gates) model failure propagation. Using fault trees one can compute dependability metrics to quantify how a system fares w.r.t.

☆ This work was partially funded by NWO, NS, and ProRail project 15474 (*SEQUOIA*), ERC grant 695614 (*POWVER*), EU project 102112 (*SUCCESS*), ANPCyT PICT-2017-3894 (*RAFTSys*), and SeCyT project 33620180100354CB (*ARES*).

The Author(s) 2020
A. Biere and D. Parker (Eds.): TACAS 2020, LNCS 12078, pp. 463–482, 2020.
https://doi.org/10.1007/978-3-030-45190-5_26

certain performance indicators. Two common metrics are system *reliability*—the probability that there are no system failures during a given mission time—and system *availability*—the average percentage of time that a system is operational.

In this paper we consider repairable dynamic fault trees. *Dynamic fault trees* (DFTs [17, 43]) are a common and widely applied variant of FTs, catering for common dependability patterns such as spare management and causal dependencies. *Repairs* [6] are not only crucial in fault-tolerant and resilient systems, they are also an important cost driver. Hence, repairable fault trees allow one to compare different repair strategies with respect to various dependability metrics.

**Fault tree analysis.** The reliability/availability of a fault tree can be computed via numerical methods, such as probabilistic model checking. This involves exhaustive explorations of state-based models such as interactive Markov chains [40]. Since the number of states (i.e. system configurations) is exponential in the number of tree elements, analysing large trees remains a challenge today [26, 1]. Moreover, numerical methods are usually restricted to exponential failure rates and combinations thereof, like Erlang and acyclic phase type distributions [40].

Alternatively, fault trees can be analysed using (standard) Monte Carlo simulation (SMC [22, 40, 38], aka statistical model checking). Here, a large number of simulated system runs (*samples*) is produced. Reliability and availability are then statistically estimated from the resulting sample set. Such sampling does not involve storing the full state space so, although the result provided can only be correct with a certain probability, SMC is much more memory efficient than numerical techniques. Furthermore, SMC is not restricted to exponential probability distributions. However, a known bottleneck of SMC are rare events: when the event of interest has a low probability (which is typically the case in highly reliable systems), millions of samples may be required to observe it. Producing these samples can take a unacceptably long simulation time.

**Rare event simulation.** To alleviate this problem, the field of rare event simulation (RES) provides techniques that reduce the number of samples [35]. The two leading techniques are importance sampling and importance splitting.

*Importance sampling* tweaks the probabilities in a model, then computes the metric of interest for the changed system, and finally adjusts the analysis results to the original model [23, 33]. Unfortunately it has specific requirements on the stochastic model: in particular, it is generally limited to Markov models.

*Importance splitting*, deployed in this paper, does not have this limitation. Importance splitting relies on rare events that arise as a sequence of less rare intermediate events [28, 2]. We exploit this fact by generating more (partial) samples on paths where such intermediate events are observed. As a simple example, consider a biased coin whose probability of heads is $p = 1/80$. Suppose we flip it eight times in a row, and say we are interested in observing at least three heads. If heads comes up at the first flip ($H$) then we are on a promising path. We can then clone (*split*) the current path $H$, generating e.g. 7 copies of it, each clone evolving independently from the second flip onwards. Say one clone observes three heads—the copied $H$ plus two more. Then, this observation of the rare event (three heads) is counted as $1/7$ rather than as 1 observation, to account for the splitting where the clone was spawned. Now, if a clone observes a new head ($HH$), this is even more promising than $H$, so the splitting can be repeated. If we make 5 copies of the $HH$ clone, then observing three heads in any of these copies counts as $\frac{1}{35} = \frac{1}{7} \cdot \frac{1}{5}$. Alternatively, observing tails as second flip ($HT$) is less promising than heads. One could then decide not to split such path.

This example highlights a key ingredient of importance splitting: the *importance function*, that indicates for each state how promising it is w.r.t. the event of interest. This function, together with other parameters such as thresholds [19], are used to choose e.g. the number of clones spawned when visiting a state. An importance function for our example could be the number of heads seen thus far. Another one could be such number, multiplied by the number of coin flips yet to come. The goal is to give *higher importance* to states from which observing the *rare event is more likely*. The efficiency of an importance splitting implementation increases as the importance function better reflects such property.

Rare event simulation has been successfully applied in several domains [34, 45, 49, 4, 5, 46]. However, a key bottleneck is that it critically relies on expert knowledge. In particular for importance splitting, finding a good importance function is a well-known highly non-trivial task [35, 25].

**Our contribution: rare event simulation for fault trees.** This paper presents an importance splitting method to analyse RFTs. In particular, we automatically derive an importance function by exploiting the description of a system as a fault tree. This is crucial, since the importance function is normally given manually in an ad hoc fashion by a domain or RES expert. We use a variety of RES algorithms based in our importance function, to estimate system unreliability and unavailability. Our approach can converge to precise estimations in increasingly reliable systems. This method has four advantages over earlier analysis methods for RFTs—which we overview in the related work section 6—namely: (1) we are able to estimate both the system reliability and availability; (2) we can handle arbitrary failure and repair distributions; (3) we can handle rare events; and (4) we can do it in a fully automatic fashion.

Technically, we build local importance functions for the (automata-semantics of the) nodes of the tree. We then aggregate these local functions into an importance function for the full tree. Aggregation uses structural induction in the layered description of the tree. Using our importance function, we implement importance splitting methods to run RES analyses. We implemented our theory in a full-stack tool chain. With it, we computed confidence intervals for the unreliability and unavailability of several case studies. Our case studies are RFTs whose failure and repair times are governed by arbitrary continuous probability density functions (PDFs). Each case study was analysed for a fixed runtime budget and in increasingly resilient configurations. In all cases our approach could estimate the narrowest intervals for the most resilient configurations.

**Paper outline.** Background on fault trees and RES is provided in Secs. 2 and 3. We detail our theory to implement RES for RFTs in Sec. 4. Using a tool chain, we performed an extensive experimental evaluation that we present in Sec. 5. We overview related work in Sec. 6 and conclude our contributions in Sec. 7.

### 2 Fault tree analysis

A fault tree '△' is a directed acyclic graph that models how component failures propagate and eventually cause the full system to fail. We consider repairable fault trees (RFTs), where failures and repairs are governed by arbitrary probability distributions.

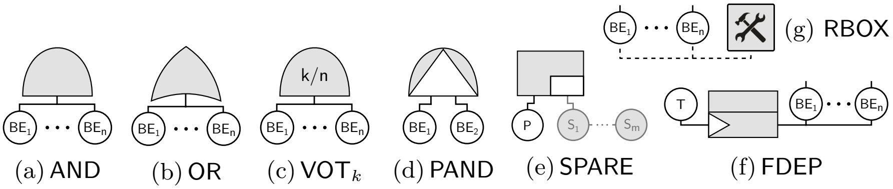

Fig. 1: Fault tree gates and the repair box

**Basic elements.** The leaves of the tree, called basic events or *basic elements* (BEs), model the failure of components. A BE $b$ is equipped with a failure distribution $F_b$ that governs the probability for $b$ to fail before time $t$, and a repair distribution $R_b$ governing its repair time. Some BEs are used as spare components: these (SBEs) replace a primary component when it fails. SBEs are equipped also with a dormancy distribution $D_b$, since spares fail less often when *dormant*, i.e. not in use. Only if an SBE becomes active, its failure distribution is given by $F_b$.

**Gates.** Non-leave nodes are called *intermediate events* and are labelled with *gates*, describing how combinations of lower failures propagate to upper levels. Fig. 1 shows their syntax. Their meaning is as follows: the AND, OR, and VOT$_k$ gates fail if respectively all, one, or $k$ of their $m$ children fail (with $1 \leqslant k \leqslant m$). The latter is called the *voting* or $k$ out of $m$ gate. Note that VOT$_1$ is equivalent to an OR gate, and VOT$_m$ is equivalent to an AND. The *priority-and gate* (PAND) is an AND gate that only fails if its children fail from left to right (or simultaneously). PANDs express failures that can only happen in a particular order, e.g. a short circuit in a pump can only occur after a leakage. SPARE gates have one *primary* child and one or more *spare* children: spares replace the primary when it fails. The FDEP gate has an input *trigger* and several *dependent events*: all dependent events become unavailable when the trigger fails. FDEPs can model for instance networks elements that become unavailable if their connecting bus fails.

**Repair boxes.** An RBOX determines which basic element is repaired next according to a given policy. Thus all its inputs are BEs or SBEs. Unlike gates, an RBOX has no output since it does not propagate failures.

**Top level event.** A full-system failure occurs if the *top event* (i.e. the root node) of the tree fails.

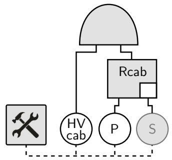

Fig. 2: Tiny RFT

**Example.** The tree in Fig. 2 models a railway-signal system, which fails if its high voltage and relay cabinets fail [21, 39]. Thus, the top event is an AND gate with children HVcab (a BE) and Rcab. The latter is a SPARE gate with primary P and spare S. All BEs are managed by one RBOX with repair priority HVcab > P > S.

**Notation.** The nodes of a tree $\triangle$ are given by $nodes(\triangle) = \{0, 1, \ldots, n-1\}$. We let $v, w$ range over $nodes(\triangle)$. A function $type^{\triangle} : nodes(\triangle) \to \{\mathsf{BE}, \mathsf{SBE}, \mathsf{AND}, \mathsf{OR}, \mathsf{VOT}_k, \mathsf{PAND}, \mathsf{SPARE}, \mathsf{FDEP}, \mathsf{RBOX}\}$ yields the type of each node in the tree. A function $chil^{\triangle} : nodes(\triangle) \to nodes(\triangle)^*$ returns the ordered list of children of a node. If clear from context, we omit the superscript $\triangle$ from function names.

**Semantics.** Following [32] we give semantics to RFT as Input/Output Stochastic Automata (IOSA), so that we can handle arbitrary probability distributions. Each state in the IOSA represents a system configuration, indicating which components are operational and which have failed. Transitions among states describe how the configuration changes when failures or repairs occur.

More precisely, a *state* in the IOSA is a tuple $\boldsymbol{x} = (\boldsymbol{x}_0, \ldots, \boldsymbol{x}_{n-1}) \in \mathcal{S} \subseteq \mathbb{N}^n$, where $\mathcal{S}$ is the *state space* and $\boldsymbol{x}_v$ denotes the state of node $v$ in $\triangle$. The possible values for $\boldsymbol{x}_v$ depend on the type of $v$. The *output* $\boldsymbol{z}_v \in \{0, 1\}$ of node $v$ indicates whether it is operational ($\boldsymbol{z}_v$=0) or failed ($\boldsymbol{z}_v$=1) and is calculated as follows:

- BEs (white circles in Fig. 1) have a binary state: $\boldsymbol{x}_v = 0$ if BE $v$ is operational and $\boldsymbol{x}_v = 1$ if it is failed. The output of a BE is its state: $\boldsymbol{z}_v = \boldsymbol{x}_v$.
- SBEs (gray circles in Fig. 1e) have two additional states: $\boldsymbol{x}_v = 2, 3$ if a *dormant* SBE $v$ is resp. operational, failed. Here $\boldsymbol{z}_v = \boldsymbol{x}_v \bmod 2$.
- ANDs have a binary state. Since the AND gate $v$ fails iff all children fail: $\boldsymbol{x}_v = \min_{w \in chil(v)} \boldsymbol{z}_w$. An AND gate outputs its internal state: $\boldsymbol{z}_v = \boldsymbol{x}_v$.
- OR gates are analogous to AND gates, but fail iff any child fail, i.e. $\boldsymbol{z}_v = \boldsymbol{x}_v = \max_{w \in chil(v)} \boldsymbol{z}_w$ for OR gate $v$.
- VOT gates also have a binary state: a $\mathsf{VOT}_k$ gate fails iff $1 \leqslant k \leqslant m$ children fail, thus $\boldsymbol{z}_v = \boldsymbol{x}_v = 1$ if $k \leqslant \sum_{w \in chil(v)} \boldsymbol{z}_w$, and $\boldsymbol{z}_v = \boldsymbol{x}_v = 0$ otherwise.
- PAND gates admit multiple states to represent the failure order of the children. For PAND $v$ with two children we let $\boldsymbol{x}_v$ equal: **0** if both children are operational; **1** if the left child failed, but the right one has not; **2** if the right child failed, but the left one has not; **3** if both children have failed, the right one first; **4** if both children have failed, otherwise. The output of PAND gate $v$ is $\boldsymbol{z}_v = 1$ if $\boldsymbol{x}_v = 4$ and $\boldsymbol{z}_v = 0$ otherwise. PAND gates with more children are handled by exploiting $\mathsf{PAND}(w_1, w_2, w_3) = \mathsf{PAND}(\mathsf{PAND}(w_1, w_2), w_3)$.
- SPARE gate $v$ leftmost input is its primary BE. All other (spare) inputs are SBEs. SBEs can be shared among SPARE gates. When the primary of $v$ fails, it is replaced with an *available* SBE. An SBE is unavailable if it is failed, or if it is replacing the primary BE of another SPARE. The output of $v$ is $\boldsymbol{z}_v = 1$ if its primary is failed and no spare is available. Else $\boldsymbol{z}_v = 0$.
- An FDEP gate has no output. All inputs are BEs and the leftmost is the trigger. We consider non-destructive FDEPs [7]: if the trigger fails, the output of all other BE is set to 1, without affecting the internal state. Since this can be modelled by a suitable combination of OR gates [32], we omit the details.

For example, the RFT from Fig. 2 starts with all operational elements, so the initial state is $\boldsymbol{x}^0 = (0, 0, 2, 0, 0)$. If then P fails, $\boldsymbol{x}_{\mathsf{P}}$ and $\boldsymbol{z}_{\mathsf{P}}$ are set to 1 (failed) and S becomes $\boldsymbol{x}_{\mathsf{S}} = 0$ (active and operational spare), so the state changes to $\boldsymbol{x}^1 = (0, 1, 0, 0, 0)$. The traces of the IOSA are given by $\boldsymbol{x}^0 \boldsymbol{x}^1 \cdots \boldsymbol{x}^n \in \mathcal{S}^*$, where a change from $\boldsymbol{x}^j$ to $\boldsymbol{x}^{j+1}$ corresponds to transitions triggered in the IOSA.

**Nondeterminism.** Dynamic fault trees may exhibit nondeterministic behaviour as a consequence of underspecified failure behaviour [15, 27]. This can happen e.g. when two SPAREs have a single shared SBE: if all elements are failed, and the SBE is repaired first, the failure behaviour depends on which SPARE gets the SBE. Monte Carlo simulation, however, requires fully stochastic models and cannot cope with nondeterminism. To overcome this problem we deploy the theory from [16, 32]. If a fault tree adheres to some mild syntactic conditions, then its IOSA semantics is *weakly deterministic*, meaning that all resolutions of the nondeterministic choices lead to the same probability value. In particular, we require that (1) each BE is connected to at most one SPARE gate, and (2) BEs and SBEs connected to SPAREs are not connected to FDEPs. In addition to this, some semantic decisions have been fixed, e.g. the semantics of PAND is fully specified, and policies should be provided for RBOX and spare assignments.

**Dependability metrics.** An important use of fault trees is to compute relevant dependability metrics. Let $X_t$ denote the random variable that represents the state of the top event at time $t$ [14]. Two popular metrics are:

– *system reliability*: the probability of observing no top event failure before some mission time $T > 0$, viz. $\mathrm{REL}_T = \mathit{Prob}\left(\forall_{t\in[0,T]} \,.\, X_t = 0\right)$;
– *system availability*: the proportion of time that the system remains operational in the long-run, viz. $\mathrm{AVA} = \lim_{t\to\infty} \mathit{Prob}\,(X_t = 0)$.

System *unreliability* and *unavailability* are the reverse of these metrics. That is: $\mathrm{UNREL}_T = 1 - \mathrm{REL}_T$ and $\mathrm{UNAVA} = 1 - \mathrm{AVA}$.

### 3 Stochastic simulation for Fault Trees

**Standard Monte Carlo simulation (SMC).** Monte Carlo simulation takes random samples from stochastic models to estimate a (dependability) metric of interest. For instance, to estimate the unreliability of a tree $\triangle$ we sample $N$ independent traces from its IOSA semantics. An unbiased statistical estimator for $p = \mathrm{UNREL}_T$ is the proportion of traces observing a top level event, that is, $\hat{p}_N = \frac{1}{N}\sum_{j=1}^{N} X^j$ where $X^j = 1$ if the $j$-th trace exhibits a top level failure before time $T$ and $X^j = 0$ otherwise. The statistical error of $\hat{p}$ is typically quantified with two numbers $\delta$ and $\varepsilon$ s.t. $\hat{p} \in [p - \varepsilon, p + \varepsilon]$ with probability $\delta$. The interval $\hat{p} \pm \varepsilon$ is called a *confidence interval* (CI) with coefficient $\delta$ and precision $2\varepsilon$.

Such procedures scale linearly with the number of tree nodes and cater for a wide range of PDFs, even non-Markovian distributions. However, they encounter a bottleneck to estimate *rare events*: if $p \approx 0$, very few traces observe $X^j = 1$. Therefore, the variance of estimators like $\hat{p}$ becomes huge, and CIs become very broad, easily degenerating to the trivial interval $[0, 1]$. Increasing the number of traces alleviates this problem, but even standard CI settings—where $\varepsilon$ is relative to $p$—require sampling an unacceptable number of traces [35]. Rare event simulation techniques solve this specific problem.

**Rare Event Simulation (RES).** RES techniques [35] increase the amount of traces that observe the rare event, e.g. a top level event in an RFT. Two prominent classes of RES techniques are *importance sampling*, which adjusts the PDF of failures and repairs, and *importance splitting* (ISPLIT [30]), which samples more (partial) traces from states that are closer to the rare event. We focus on ISPLIT due to its flexibility with respect to the probability distributions.

ISPLIT can be efficiently deployed as long as the rare event $\gamma$ can be described as a nested sequence of less-rare events $\gamma = \gamma_M \subsetneq \gamma_{M-1} \subsetneq \cdots \subsetneq \gamma_0$. This decomposition allows ISPLIT to study the conditional probabilities $p_k = \mathit{Prob}(\gamma_{k+1} \mid \gamma_k)$ separately, to then compute $p = \mathit{Prob}(\gamma) = \prod_{k=0}^{M\text{-}1} \mathit{Prob}(\gamma_{k+1} \mid \gamma_k)$. Moreover, ISPLIT requires all conditional probabilities $p_k$ to be much greater than $p$, so that estimating each $p_k$ can be done efficiently with SMC.

The key idea behind ISPLIT is to define the events $\gamma_k$ via a so called *importance function* $\mathcal{I}\colon \mathcal{S} \to \mathbb{N}$ that assigns an *importance* to each state $s \in \mathcal{S}$. The higher the importance of a state, the closer it is to the rare event $\gamma_M$. Event $\gamma_k$ collects all states with importance at least $\ell_k$, for certain sequence of *threshold levels* $0 = \ell_0 < \ell_1 < \cdots < \ell_M$. Formally: $\gamma_k = \{s \in \mathcal{S} \mid \mathcal{I}(s) \geqslant \ell_k\}$.

To exploit the importance function $\mathcal{I}$ in the simulation procedure, ISPLIT samples more (partial) traces from states with higher importance. Two well-known methods are deployed and compared in this paper: Fixed Effort and RESTART. *Fixed Effort* (FE [19]) samples a predefined amount of traces in each region $\mathcal{S}_k = \gamma_k \setminus \gamma_{k+1} = \{s \in \mathcal{S} \mid \ell_{k+1} > \mathcal{I}(s) \geqslant \ell_k\}$. Thus, starting at $\gamma_0$ it first estimates the proportion of traces that reach $\gamma_1$, i.e. $p_0 = \mathit{Prob}(\gamma_1 \mid \gamma_0) = \mathit{Prob}(\mathcal{S}_0)$. Next, from the states that reached $\gamma_1$ new traces are generated to estimate $p_1 = \mathit{Prob}(\mathcal{S}_1)$, and so on until $p_M$. Fixed Effort thus requires that (*i*) each trace has a clearly defined "end," so that estimations of each $p_k$ finish with probability 1, and (*ii*) all rare events reside in the uppermost region.

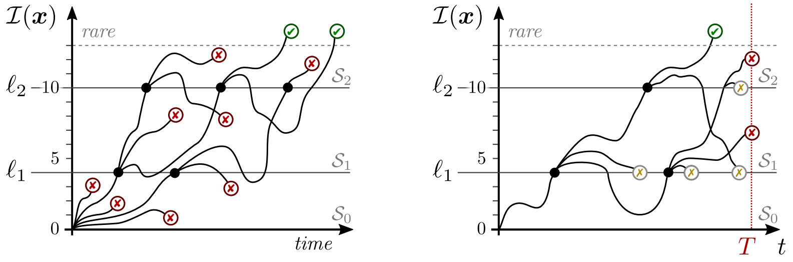

(a) $\mathrm{FE}_5$ for $\mathit{Prob}(\neg ✘ \,\mathsf{U}\, ✔)$ (b) $\mathrm{RST}_{\mathrm{ES}}$ for $\mathrm{UNREL}_T$

Fig. 3: Importance Splitting algorithms Fixed Effort & RESTART

**Example.** Fig. 3a shows Fixed Effort estimating the probability to visit states labelled ✔ before others labelled ✘. States ✔ have importance >13, and thresholds $\ell_1, \ell_2 = 4, 10$ partition the state space in regions $\{\mathcal{S}_i\}_{i=0}^2$ s.t. all ✔ $\in \mathcal{S}_2$. The effort is 5 simulations per region, for all regions: we call this algorithm $\mathrm{FE}_5$.

In region $\mathcal{S}_0$, 2 simulations made it from the initial state to threshold $\ell_1$, i.e. they reached some state with importance 4 before visiting a state ✘. In $\mathcal{S}_1$, starting from these two states, 3 simulations reached $\ell_2$. Finally, 2 out of 5 simulations visited states ✔ in $\mathcal{S}_2$. Thus, the estimated rare event probability of this run of FE 5 is $\hat{p} = \prod_{i=1}^{2} \hat{p_i} = \frac{2}{5}\frac{3}{5}\frac{2}{5} = 9.6 \times 10^{-2}$.

*RESTART* (RST [48, 47]) is another RES algorithm, which starts one trace in $\gamma_0$ and monitors the importance of the states visited. If the trace up-crosses threshold $\ell_1$, the first state visited in $\mathcal{S}_1$ is saved and the trace is cloned, aka *split*—see Fig. 3b. This mechanism rewards traces that get closer to the rare event. Each clone then evolves independently, and if one up-crosses threshold $\ell_2$ the splitting mechanism is repeated. Instead, if a state with importance below $\ell_1$ is visited, the trace is *truncated* (ⓧ in Fig. 3b). This penalises traces that move away from the rare event. To avoid truncating all traces, the one that spawned the clones in region $\mathcal{S}_k$ can go below importance $\ell_k$. To deploy an unbiased estimator for $p$, RESTART measures how much split was required to visit a rare state [47]. In particular, RESTART does not need the rare event to be defined as $\gamma_M$ [44], and it was devised for steady-state analysis [48] (e.g. to estimate UNAVA) although it can also been used for transient studies as depicted in Fig. 3b [45].

### 4 Importance Splitting for FTA

The effectiveness of ISPLIT crucially relies on the choice of the importance function $\mathcal{I}$ as well as the threshold levels $\ell_k$ [30]. Traditionally, these are given by domain and/or RES experts, requiring a lot of domain knowledge. This section presents a technique to obtain $\mathcal{I}$ and the $\ell_k$ automatically for an RFT.

#### 4.1 Compositional importance functions for Fault Trees

By the core idea behind importance splitting, states that are more likely to lead to the rare event should have a higher importance. To achieve this, the key lies in defining an importance function $\mathcal{I}$ and thresholds $\ell_k$ that are sensitive to both the state space $\mathcal{S}$ and the transition probabilities of the system. For us, $\mathcal{S} \subseteq \mathbb{N}^n$ are all possible states of a repairable fault tree (RFT). Its top event fails when certain nodes fail in certain order, and remain failed before certain repairs occur. To exploit this for ISPLIT, the structure of the tree must be embedded into $\mathcal{I}$.

The strong dependence of the importance function $\mathcal{I}$ on the structure of the tree is easy to see in the following example. Take the RFT $\triangle$ from Fig. 2 and let its current state $\boldsymbol{x}$ be s.t. P is failed and HVcab and S are operational. If the next event is a repair of P, then the new state $\boldsymbol{x}'$ (where all basic elements are operational) is farther from a failure of the top event. Hence, a good importance function should satisfy $\mathcal{I}(\boldsymbol{x}) > \mathcal{I}(\boldsymbol{x}')$. Oppositely, if the next event had been a failure of S leading to state $\boldsymbol{x}''$, then one would want that $\mathcal{I}(\boldsymbol{x}) < \mathcal{I}(\boldsymbol{x}'')$. The key observation is that these inequalities depend on the structure of $\triangle$ as well as on the failures/repairs of basic elements.

In view of the above, any attempt to define an importance function for an arbitrary fault tree $\triangle$ must put its gate structure in the forefront. In Table 1 we introduce a compositional heuristic for this, which defines *local importance functions* distinguished per node type. The importance function associated to node $v$ is $\mathcal{I}_v \colon \mathbb{N}^n \to \mathbb{N}$. We define the *global importance function* of the tree ($\mathcal{I}_\triangle$ or simply $\mathcal{I}$) as the local importance function of the top event node of $\triangle$.

Table 1: Compositional importance function for RFTs

| *type*$(v)$ | $\mathcal{I}_v(\boldsymbol{x})$ |
|---|---|
| BE, SBE | $\boldsymbol{z}_v$ |
| AND | $\mathrm{lcm}_v \cdot \sum_{w \in chil(v)} \frac{\mathcal{I}_w(\boldsymbol{x})}{\max^{\mathcal{I}}_w}$ |
| OR | $\mathrm{lcm}_v \cdot \max\limits_{w \in chil(v)} \left\{ \frac{\mathcal{I}_w(\boldsymbol{x})}{\max^{\mathcal{I}}_w} \right\}$ |
| VOT$_k$ | $\mathrm{lcm}_v \cdot \max\limits_{W \subseteq chil(v), \lvert W \rvert = k} \left\{ \sum_{w \in W} \frac{\mathcal{I}_w(\boldsymbol{x})}{\max^{\mathcal{I}}_w} \right\}$ |
| SPARE | $\mathrm{lcm}_v \cdot \max \left( \sum_{w \in chil(v)} \frac{\mathcal{I}_w(\boldsymbol{x})}{\max^{\mathcal{I}}_w} \;,\; \boldsymbol{z}_v \cdot m \right)$ |
| PAND | $\mathrm{lcm}_v \cdot \max \left( \frac{\mathcal{I}_l(\boldsymbol{x})}{\max^{\mathcal{I}}_l} + ord\, \frac{\mathcal{I}_r(\boldsymbol{x})}{\max^{\mathcal{I}}_r} \;,\; \boldsymbol{z}_v \cdot 2 \right)$ where $ord = 1$ if $\boldsymbol{x}_v \in \{1, 4\}$ and $ord = -1$ otherwise |
| with $\max^{\mathcal{I}}_v = \max_{\boldsymbol{x} \in \mathcal{S}} \mathcal{I}_v(\boldsymbol{x})$ and $\mathrm{lcm}_v = \mathrm{lcm}\left\{ \max^{\mathcal{I}}_w \,\middle|\, w \in chil(v) \right\}$ | |

Thus, $\mathcal{I}_v$ is defined in Table 1 via structural induction in the fault tree. It is defined so that it assigns to a *failed* node $v$ its *highest importance value*. Functions with this property deploy the most efficient ISPLIT implementations [30], and some RES algorithms (e.g. Fixed Effort) require this property [19].

In the following we explain our definition of $\mathcal{I}_v$. If $v$ is a failed BE or SBE, then its importance is 1; else it is 0. This matches the output of the node, thus $\mathcal{I}_v(\boldsymbol{x}) = \boldsymbol{z}_v$. Intuitively, this reflects how failures of basic elements are positively correlated to top event failures. The importance of AND, OR, and VOT$_k$ gates depends exclusively on their input. The importance of an AND is the sum of the importance of their children scaled by a normalisation factor. This reflects that AND gates fail when all their children fail, and each failure of a child brings an AND closer to its own failure, hence increasing its importance. Instead, since OR gates fail as soon as a single child fails, their importance is the maximum importance among its children. The importance of a VOT$_k$ gate is the sum of the $k$ (out of $m$) children with highest importance value.

Omiting normalisation may yield an undesirable importance function. To understand why, suppose a binary AND gate $v$ with children $l$ and $r$, and define $\mathcal{I}^{\text{naive}}_v(\boldsymbol{x}) = \mathcal{I}_l(\boldsymbol{x}) + \mathcal{I}_r(\boldsymbol{x})$. Suppose that $\mathcal{I}_l$ takes it highest value in $\max^{\mathcal{I}}_l = 2$ while $\mathcal{I}_r$ in $\max^{\mathcal{I}}_r = 6$ and assume that states $\boldsymbol{x}$ and $\boldsymbol{x}'$ are s.t. $\mathcal{I}_l(\boldsymbol{x}) = 1$,

$\mathcal{I}_r(\boldsymbol{x}) = 0$, $\mathcal{I}_l(\boldsymbol{x}') = 0$, $\mathcal{I}_r(\boldsymbol{x}') = 3$. This means that in both states one child of $v$ is "good-as-new" and the other is "half-failed" and hence the system is equally close to fail in both cases. Hence we expect $\mathcal{I}_v^{\text{naive}}(\boldsymbol{x}) = \mathcal{I}_v^{\text{naive}}(\boldsymbol{x}')$ when actually $\mathcal{I}_v^{\text{naive}}(\boldsymbol{x}) = 1 \neq 3 = \mathcal{I}_v^{\text{naive}}(\boldsymbol{x}')$. Instead, $\mathcal{I}_v$ operates with $\frac{\mathcal{I}_l(\boldsymbol{x})}{\max_l^{\mathcal{I}}}$ and $\frac{\mathcal{I}_r(\boldsymbol{x})}{\max_r^{\mathcal{I}}}$, which can be interpreted as the "percentage of failure" of the children of $v$. To make these numbers integers we scale them by $\text{lcm}_v$, the *least common multiple* of their max importance values. In our case $\text{lcm}_v = 6$ and hence $\mathcal{I}_v(\boldsymbol{x}) = \mathcal{I}_v(\boldsymbol{x}') = 3$. Similar problems arise whit all gates, hence normalization is applied in general.

SPARE gates with $m$ children (including its primary) behave similarly to AND gates: every failed child brings the gate closer to failure, as reflected in the left operand of the max in Table 1. However, SPAREs fail when their primaries fail and no SBEs are *available*, e.g. possibly being used by another SPARE. This means that the gate could fail in spite of some children being operational. To account for this we exploit the gate output: multiplying $\boldsymbol{z}_v$ by $m$ we give the gate its maximum value when it fails, even when this happens due to unavailable but operational SBEs. For a PAND gate $v$ we have to carefully look at the states. If the left child $l$ has failed, then the right child $r$ contributes positively to the failure of the PAND and hence the importance function of the node $v$. If instead the right child has failed first, then the PAND gate will not fail and hence we let it contribute negatively to the importance function of $v$. Thus, we multiply $\frac{\mathcal{I}_r(\boldsymbol{x})}{\max_r^{\mathcal{I}}}$ (the normalized importance function of the right child) by $-1$ in the later case (i.e. when state $\boldsymbol{x}_v \notin \{1, 4\}$). Instead, the left child always contribute positively. Finally, the max operation is two-fold: on the one hand, $\boldsymbol{z}_v \cdot 2$ ensures that the importance value remains at its maximun while failing (PANDs remain failed even after the left child is repaired); on the other, it ensures that the smallest value posible is 0 while operational (since importance values can not be negative.)

#### 4.2 Automatic importance splitting for FTA

Our compositional importance function is based on the distribution of operational/failed basic elements in the fault tree, and their failure order. This follows the core idea of importance splitting: the more failed BEs/SBEs (in the right order), the closer a tree is to its top event failure.

However, ISPLIT is about running more simulations from state with higher *probability* to lead to rare states. This is only partially reflected by whether basic element $b$ is failed. Probabilities lie also in the distributions $F_b, R_b, D_b$. These distributions govern the transitions among states $\boldsymbol{x} \in \mathcal{S}$, and can be exploited for importance splitting. We do so using the two-phased approach of [11, 12], which in a first (static) phase computes an importance function, and in a second (dynamic) phase selects the thresholds from the resulting importance values.

In our current work, the first phase runs breadth-first search in the IOSA module of each tree node. This computes node-local importance functions, that are aggregated into a tree-global $\mathcal{I}$ using our compositional function in Table 1.

The second phase involves running "pilot simulations" on the importance-labelled states of the tree. Running simulations exercises the fail/repair distributions of BEs/SBEs, imprinting this information in the thresholds $\ell_k$. Several algorithms can do such *selection of thresholds*. They operate sequentially, starting from the initial state—a fully operational tree—which has importance $i_0 = 0$. For instance, Expected Success [10] runs $N$ finite-life simulations. If $K < \frac{N}{2}$ simulations reach the next smallest importance $i_1 > i_0$, then the first threshold will be $\ell_1 = i_1$. Next, $N$ simulations start from states with importance $i_1$, to determine whether the next importance $i_2$ should be chosen as threshold $\ell_2$, and so on.

Expected Success also computes the *effort* per splitting region $\mathcal{S}_k = \{\boldsymbol{x} \in \mathcal{S} \mid \ell_{k+1} > \mathcal{I}(\boldsymbol{x}) \geqslant \ell_k\}$. For Fixed Effort, "effort" is the base number of simulations to run in region $\mathcal{S}_k$. For RESTART, it is the number of clones spawned when threshold $\ell_{k+1}$ is up-crossed. In general, if $K$ out of $N$ pilot simulations make it from $\ell_{k-1}$ to $\ell_k$, then the $k$-th effort is $\lceil \frac{N}{K} \rceil$. This is chosen so that, during RES estimations, one simulation makes it from threshold $\ell_{k-1}$ to $\ell_k$ on average.

Thus, using the method from [11, 12] based on our importance function $\mathcal{I}_\triangle$, we compute (automatically) the thresholds and their effort for tree $\triangle$. This is all the meta-information required to apply importance splitting RES [19, 18, 11].

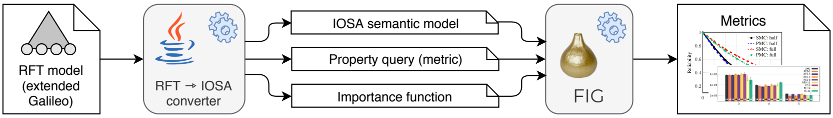

Fig. 4: Tool chain

**Implementation.** Fig. 4 outlines a tool chain implemented to deploy the theory described above. The input model is an RFT, described in the Galileo textual format [42, 41] extended with repairs and arbitrary PDFs. This RFT file is given as input to a Java converter that produces three outputs: the IOSA semantics of the tree, the property queries for its reliability or availability, and our compositional importance function in terms of variables of the IOSA semantic model. This information is dumped into a single text file and fed to FIG: a statistical model checker specialised in importance splitting RES. FIG interprets this importance function, deploying it into its internal model representation, which results in a global function for the whole tree. FIG can then use ISPLIT algorithms such as RESTART and Fixed Effort, via the automatic methods described above. The result are confidence intervals that estimate the reliability or availability of the RFT. In this way, we implemented automatic importance splitting for FTA. In [9] we provide more details about our tool chain and its capabilities.

## 5 Experimental evaluation

### 5.1 General setup

Using our tool chain, we computed the unreliability and unavailability of 26 highly-resilient repairable non-Markovian DFTs. These trees come from seven literature case studies, enriched with RBOX elements and non-Markovian PDFs. We estimated their $\mathrm{UNREL}_{10^3}$ or UNAVA in increasingly resilient configurations.

To estimate these values we used various simulation algorithms: Standard Monte Carlo (SMC); Fixed Effort [19] for different number of runs performed in each $\mathcal{S}_k$ region ($\mathrm{FE}_n$ for $n = 8, 12, 16, 24, 32$); RESTART [47] with thresholds selected via a Sequential Monte Carlo algorithm [12] for different global splitting values ($\mathrm{RST}_n$ for $n = 2, 3, 5, 8, 11$); and RESTART with thresholds selected via Expected Success [10], which computes splitting values independently for each threshold ($\mathrm{RST}_{\mathrm{ES}}$). $\mathrm{FE}_n$, $\mathrm{RST}_n$, and $\mathrm{RST}_{\mathrm{ES}}$, used the automatic ISPLIT framework based in our importance function, as described in Sec. 4.2.

An *instance* $\mathfrak{y}$ is a combination of an algorithm *algo*, an RFT, and a dependability metric. An RFT is identified by a case study ($\mathcal{cs}$) and a parameter ($\mathfrak{p}$), where larger parameters of the RFT $\mathcal{cs}_{\mathfrak{p}}$ indicate smaller dependability values $p_{\mathcal{cs}_{\mathfrak{p}}}$. Running *algo* for a fixed simulation time, instance $\mathfrak{y}$ estimates the value $p_{\mathfrak{y}} = p_{\mathcal{cs}_{\mathfrak{p}}}$. The resulting CI ($\hat{p}_{\mathfrak{y}}$) has a certain width $\|\hat{p}_{\mathfrak{y}}\| \in [0, 1]$ (we fix the confidence coefficient $\delta = 0.95$). The performance of *algo* can be measured by that width: the smaller $\|\hat{p}_{\mathfrak{y}}\|$, the more efficient the algorithm that achieved it.

The simulation time fixed for an RFT may not suffice to observe rare events, e.g. for SMC. In such cases the FIG tool reports a "null estimate" $\hat{p}_{\mathfrak{y}} = [0, 0]$. Moreover, the simulation of random events depends on the RNG—and its seed—used by FIG, so different runs may yield different results $\hat{p}_{\mathfrak{y}}$. Therefore, for each $\mathfrak{y}$ we repeated $n = 10$ times the computation of $\hat{p}_{\mathfrak{y}}$, to assess the performance of *algo* in $\mathfrak{y}$ by: (*i*) how many times it yielded not-null estimates, indicated with a bold number at the base of the bar corresponding to $\mathfrak{y}$ (e.g. **8** **10** in Fig. 5b); (*ii*) what was the average width $\|\hat{p}_{\mathfrak{y}}\|$, using not-null estimates only, indicated by the height of the bar; and (*iii*) what was the standard deviation of those widths, indicated by whiskers on top of the bar. We performed $n = 10$ repetitions to ensure statistical significance: a 95% CI for a plotted bar is narrower than the whiskers and, in the hardest configuration of every $\mathcal{cs}$, the whiskers of SMC bars never overlap with those of the best RES algorithm.

**Case studies.** Our seven parametric case studies are: the synthetic models $\mathrm{DSPARE}_n$ and $\mathrm{VOT}_m$, with $n \in \{3, 4, 5\}$ SBEs the first, $m \in \{2, 3, 4\}$ shared BEs the second, and one RBOX each; $\mathrm{FTPP}_s$ [17], where we study one triad with $s \in \{4, 5, 6\}$ shared SBEs, using one RBOX for the processors and another for the network elements; $\mathrm{HECS}_o$ [43], with 2 memory interfaces, 4 RBOX (one per subsystem), $o \in \{1, \ldots, 5\}$ shared spare processors, and $2o$ parallel buses; and $\mathrm{RWC}_{u \in \{4, \ldots, 7\}}$ [22, 21, 39], which combines subsystems $\mathrm{RC}_v$ with one RBOX and $v \in \{3, \ldots, 6\}$ SPAREs, and $\mathrm{HVC}_w$ with another RBOX and $w \in \{2, \ldots, 4\}$ shared SBEs. In total these are 26 RFTs with PDFs that include exponential, Erlang, uniform, Rayleigh, Weibull, normal, and log-normal distributions. In an extended version of this work [9] we provide all details of our case studies.

**Hardware.** Experiments ran in two types of nodes in a SLURM cluster running Linux x64 (Ubuntu, kernel 3.13.0-168): *korenvliet* nodes have CPUs Intel® Xeon® E5-2630 v3 @ 2.40 GHz, and 64 GB of DDR4 RAM @ 1600 MHz; *caserta* has CPUs Intel® Xeon® E7-8890 v4 @ 2.20 GHz, and 2 TB of RAM DDR4 @ 1866 MHz.

### 5.2 Experimental results and discussion

Using SMC and RESTART we computed UNAVA for $\text{VOT}_{2,3,4}$, $\text{HECS}_{1,\ldots,5}$, $\text{RC}_{3,\ldots,6}$, and $\text{RWC}_{1,\ldots,4}$. FE was not used since it requires regeneration theory for steady-state analysis [19], which is not always feasible with non-Markovian models. The mean widths of the CIs achieved per instance are shown in Fig. 5.

For example for $\text{VOT}_2$ (Fig. 5a), 10 independent computations with SMC ran in caserta for 5 min, and all converged to not-null CIs ( **10** ). The mean width of these CIs was $1.40{\times}10^{-4}$ and their standard deviation $7.96{\times}10^{-6}$. For $\text{VOT}_3$, all SMC computations yielded not-null CIs (after 30 min) with an average precision of $9.62{\times}10^{-6}$ and standard deviation $1.52{\times}10^{-6}$. For $\text{VOT}_4$ all SMC simulations yielded null CIs after 3 hours of simulation (**0**). Instead, $\text{RST}_2$ converged to 10, 10, and 5 not-null CIs resp. for $\text{VOT}_{2,3,4}$, with mean widths (and standard deviation): $1.24{\times}10^{-4}$ ($1.19{\times}10^{-5}$), $5.09{\times}10^{-6}$ ($1.48{\times}10^{-6}$), and $1.79{\times}10^{-7}$ ($3.19{\times}10^{-8}$). Thus for the VOT case study, $\text{RST}_2$ was consistently more efficient than SMC, and the efficiency gap increased as UNAVA became rarer.

This trend repeats in all experiments: as expected, the rarer the metric, the wider the CIs computed in the time limit, until at some point it becomes very hard to converge to not-null CIs at all (specially for SMC). For the least resilient configuration of each case study, SMC can be competitive or even more efficient than some ISPLIT variants. For instance for $\text{VOT}_1$ and $\text{HECS}_1$ in Figs. 5a and 5b, all computations converged to not-null CIs for all algorithms, but SMC exhibits less variable CI widths, viz. smaller whiskers. This is reasonable: truncating and splitting traces in RESTART adds $(i)$ simulation overhead that may not pay off to estimate not-so-rare events, and on top of it $(ii)$ correlations of cloned traces that share a common history, increasing the variability among independent runs. On the other hand and as expected, SMC looses this competitiveness for all case studies as failures become rarer, here when UNAVA $\leqslant 1.0{\times}10^{-5}$. This

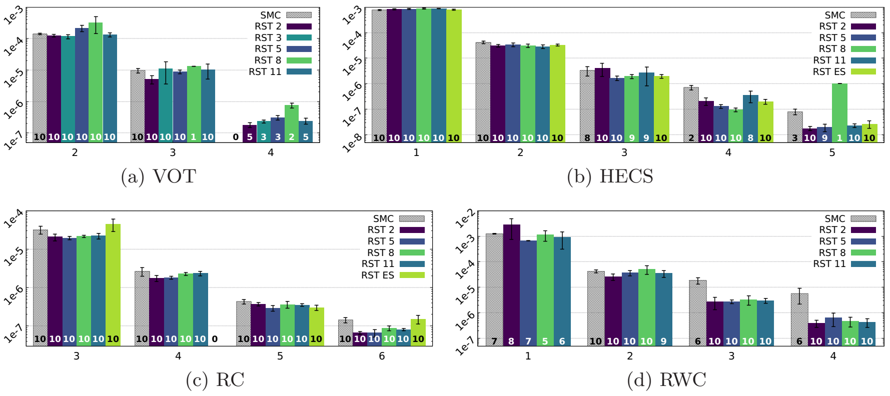

Fig. 5: CI precision for system unavailability holds nicely for the biggest case studies: HECS$_5$[†] (a 42-nodes RFT whose IOSA has 126-not-clock variables $\approx 2.89{\times}10^{38}$ states, with 57 clocks of exponential, uniform, and log-normal PDFs) and RWC$_4$ (42 nodes, 181 variables $\approx 6.93{\times}10^{73}$ states, 62 clocks of exponential, Erlang, Rayleigh, uniform, and normal PDFs).

Using SMC, RESTART, and FE, we also estimated UNREL$_{1000}$ for RWC$_{2,3,4}$, DSPARE$_{3,4,5}$, FTPP$_{4,5,6}$, HVC$_{4,5,6,7}$, and HECS$_{2,3,4,5}$. For HVC (only) we ran 20 experiments per tree, 10 in each cluster node. Fig. 6 shows the results.

Fig. 6: CI precision for system unreliability

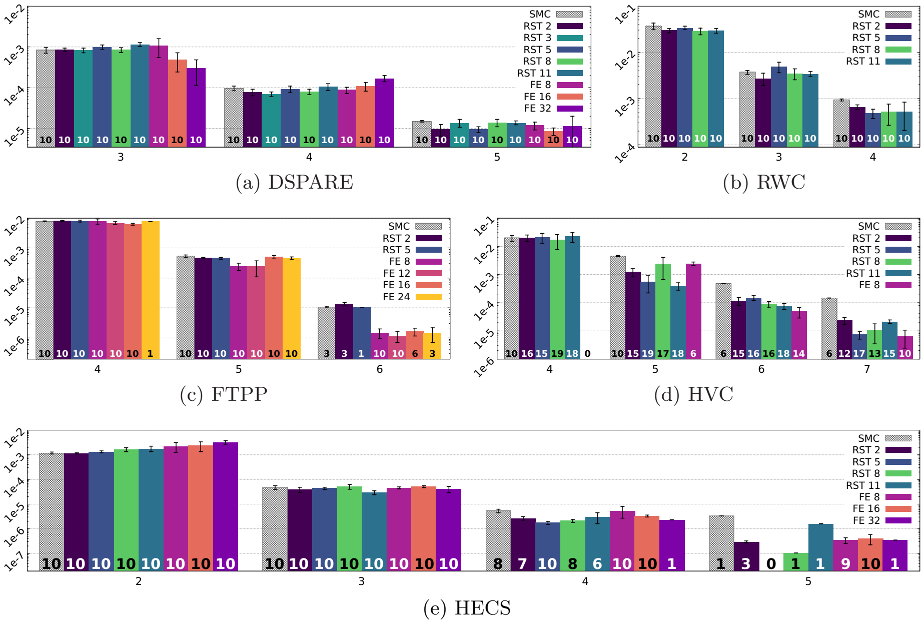

The overall trend shown for unreliability estimations is similar to the previous unavailability cases. Here however it was possible to use Fixed Effort, since every simulation has a clearly defined end at time $T = 10^3$. It is interesting thus to compare the efficiency of RESTART vs. FE: we note for example that some variants of FE performed considerably better than any other approach in the most resilient configurations of FTPP and HECS. It is nevertheless difficult to draw general conclusions from Figs. 6a to 6e, since some variants that performed best in a case study—e.g. FE$_{16}$ in HECS—did worse in others—e.g. FTPP, where the best algorithms were FE$_{8,12}$. Furthermore, FE$_8$, which is always better than

† RST$_8$ for HECS$_5$ escapes this trend: analysing the execution logs it was found that FIG crashed during the second computation.

SMC when $\text{UNREL}_{1000} < 10^{-3}$, did not perform very well in HVC, where the algorithms that achieved the narrowest and most not-null CIs were $\text{RST}_{5,11}$. Such cases notwithstanding, FE is a solid competitor of RESTART in our benchmark.

Another relevant point of study is the optimal effort $e$ for $\text{RST}_\text{e}$ or $\text{FE}_\text{e}$, which shows no clear trend in our experiments. Here, $e$ is a "global effort" used by these algorithms, equal for all $\mathcal{S}_k$ regions. $e$ also alters the way in which the thresholds selection algorithm Sequential Monte Carlo (SEQ [12]) selects the $\ell_k$. The lack of guidelines to select a value for $e$ that works well across different systems was raised in [8]. This motivated the development of Expected Success (ES [10]), which selects efforts individually per $\mathcal{S}_k$ (or $\ell_k$). Thus, in $\text{RST}_\text{ES}$, a trace up-crossing threshold $\ell_k$ is split according to the individual effort $e_k$ selected by ES. In the benchmark of [10], which consists mostly of queueing systems, ES was shown superior to SEQ. However, experimental outcomes on DFTs in this work are different: for UNAVA, $\text{RST}_\text{ES}$ yielded mildly good results for HECS and RC; for the other case studies and for all $\text{UNREL}_{1000}$ experiments, $\text{RST}_\text{ES}$ always yielded null CIs. It was found that the effort selected for most thresholds $\ell_k$ was either too small—so splitting in $e_k$ was not enough for the $\text{RST}_\text{ES}$ trace to reach $\ell_{k+1}$—or too large—so there was a splitting/truncation overhead. This point is further addressed in the conclusions.

Beyond comparisons among the specific algorithms, be these for RES or for selecting thresholds, it seems clear that our approach to FTA via ISPLIT deploys the expected results. For each parameterised case study $cs_\mathfrak{p}$, we could find a value of the parameter $\mathfrak{p}$ where the level of resilience is such, that SMC is less efficient than our automatically-constructed ISPLIT framework. This is particularly significant for big DFTs like HECS and RWC, whose complex structure could be exploited by our importance function.

### 6 Related work

Most work on DFT analysis assumes discrete [43, 3] or exponentially distributed [15, 29] components failure. Furthermore, components repair is seldom studied in conjunction with dynamic gates [6, 3, 40, 29, 31]. In this work we address repairable DFTs, whose failure and repair times can follow arbitrary PDFs. More in detail, RFTs were first formally introduced as stochastic Petri nets in [6, 13]. Our work stands on [32], which reviews [13] in the context of stochastic automata with arbitrary PDFs. In particular we also address non-Markovian continuous distributions: in Sec. 5 we experimented with exponential, Erlang, uniform, Rayleigh, Weibull, normal, and log-normal PDFs. Furthermore and for the first time, we consider the application of [13, 32] to study rare events.

Much effort in RES has been dedicated to study highly reliable systems, deploying either importance splitting or sampling. Typically, importance sampling can be used when the system takes a particular shape. For instance, a common assumption is that all failure (and repair) times are exponentially distributed with parameters $\lambda^i$, for some $\lambda \in \mathbb{R}$ and $i \in \mathbb{N}_{>0}$. In these cases, a favourable change of measure can be computed analytically [20, 23, 33, 34, 49, 39].

In contrast, when the fail/repair times follow less-structured distributions, importance splitting is more easily applicable. As long as a full system failure can be broken down into several smaller components failures, an importance splitting method can be devised. Of course, its efficiency relies heavily on the choice of importance function. This choice is typically done ad hoc for the model under study [44, 30, 46]. In that sense [24, 25, 11, 12] are among the first to attempt a heuristic derivation of all parameters required to implement splitting. This is based on formal specifications of the model and property query (the dependability metric). Here we extended [11, 12, 8], using the structure of the fault tree to define composition operands. With these operands we aggregate the automatically-computed local importance functions of the tree nodes. This aggregation results in an importance function for the whole model.

### 7 Conclusions

We have presented a theory to deploy *automatic importance splitting* (ISPLIT) for fault tree analysis of repairable dynamic fault trees (RFTs). This Rare Event Simulation approach supports arbitrary probability distributions of components failure and repair. The core of our theory is an importance function $\mathcal{I}_{\triangle}$ defined structurally on the tree. From such function we implemented ISPLIT algorithms, and used them to estimate the *unreliability* and *unavailability* of highly-resilient RFTs. Departing from classical approaches, that define importance functions ad hoc using expert knowledge, our theory computes all metadata required for RES from the model and metric specifications. Nonetheless, we have shown that for a fixed simulation time budget and in the most resilient RFTs, diverse ISPLIT algorithms can be automatically implemented from $\mathcal{I}_{\triangle}$, and always converge to narrower confidence intervals than standard Monte Carlo simulation.

There are several paths open for future development. First and foremost, we are looking into new ways to define the importance function, e.g. to cover more general categories of FTs such as fault maintenance trees [37]. It would also be interesting to look into possible correlations among specific RES algorithms and tree structures, that yield the most efficient estimations for a particular metric. Moreover, we have defined $\mathcal{I}_{\triangle}$ based on the tree structure alone. It would be interesting to further include stochastic information in this phase, and not only afterwards during the thresholds-selection phase.

Regarding thresholds, the relatively bad performance of the Expected Success algorithm shows a spot for improvement. In general, we believe that enhancing its statistical properties should alleviate the behaviour mentioned in Sec. 5.2. Moreover, techniques to increase trace independence during splitting (e.g. resampling) could further improve the performance of the ISPLIT algorithms. Finally, we are investigating enhancements in IOSA and our tool chain, to exploit the ratio between fail and dormancy PDFs of SBEs in warm SPARE gates.

**Acknowledgments**

The authors thank José and Manuel Villén-Altamirano, for fruitful discussions that helped to better understand the application scope of our approach.
